# Immunohistochemical expression of NF-κB signaling pathway components in the septum of type I intestinal atresia

**DOI:** 10.3389/fped.2026.1978002

**Published:** 2026-10-19

**Authors:** Xuelai Liu, Huan Wang, Yuqiang Chen, Mao Ye, Xiaoyun Cui, Yandong Wei, Feng He

**Affiliations:** 1 Department of General Surgery, Capital Center for Children’s Health, Capital Medical University, Capital Institute of Pediatrics, Beijing, China; 2 Department of Pathology, Beijing Children’s Hospital Heilongjiang Hospital, Harbin, China; 3 Department of Science and Technology, Capital Center for Children's Health, Capital Medical University, Capital Institute of Pediatrics, Beijing, China; 4 Department of Neonatal Surgery, Capital Center for Children’s Health, Capital Medical University, Capital Institute of Pediatrics, Beijing, China; 5 Department of Biochemistry and Immunology, Capital Center for Children's Health, Capital Medical University, Capital Institute of Pediatrics, Beijing, China

**Keywords:** expression, intestinal atresia, NF-*κ*B, septum, signaling pathway

## Abstract

**Background:**

The aim of the study was to characterize the expression of nuclear factor (NF)-*κ*B signaling pathway molecules in the septal mucosa of type I intestinal atresia (IA) and to explore their potential association with impaired intestinal recanalization during embryonic development.

**Methods:**

Septa and adjacent proximal intestinal wall tissue were obtained from eight patients with type I IA. Serial sections were prepared for immunohistochemistry staining of transforming growth factor-β-activated kinase 1 (TAK1), I kappa B kinase-alpha/beta (IKK-α/β), p65 NF-*κ*B, tumor necrosis factor-alpha (TNF-α), and interleukin-6 (IL-6). Mean optical density was quantified using Image-Pro Plus software, and differences between groups were statistically analyzed.

**Results:**

TAK1, IKK-α/β, and p65 NF-*κ*B showed weak cytoplasmic immunoreactivity in the glandular epithelial cells of the control intestinal mucosa. In contrast, markedly increased cytoplasmic staining for TAK1, IKK-α/β, and p65 NF-*κ*B proteins was observed in the mucosal glands of septa. The expression of TNF-α and IL-6, typical downstream pro-inflammatory cytokines of the NF-*κ*B signaling pathway, demonstrated markedly increased cytoplasmic staining. A semiquantitative analysis confirmed a significantly higher expression of TAK1, IKK-α/β, p65 NF-*κ*B, TNF-α, and IL-6 in septa than in the normal intestinal wall.

**Conclusion:**

The aberrant expression of the NF-*κ*B signaling pathway and activation of the inflammatory response during embryonic intestinal recanalization may be associated with the formation and progression of septa.

## 1 Introduction

Intestinal atresia (IA) is a common cause of neonatal intestinal obstruction (1–4). Failure of recanalization during the transient solid stage of embryonic intestinal development has been proposed as one mechanism underlying this condition (5–7). Our previous study has shown that the gut lumen contains numerous vacuoles of different sizes and that the recanalization process occurs in human embryos and fetuses through histological examination and 3D reconstruction (8). Moreover, through the evaluation of lumen development, we have demonstrated the recanalization process, in which some vacuoles in the mesenchyme of the gut merge with the developing mucosal epithelium from the proximal to the distal intestine. Based on this, we proposed that the potential morphological mechanism of failure of recanalization caused by the septum correlates with the residual vacuoles during gut lumen development (9). During gestation, the fetal gut is normally devoid of bacteria. This sterility results from the surrounding amniotic fluid, which contains multiple antimicrobial agents that hinder microbial invasion, and from the placental barrier, which supplies oxygen and nutrients, eliminates metabolic waste, and prevents most pathogens and toxins from reaching the fetus. Hence, in healthy circumstances, the fetal gut remains bacteria-free (10, 11). Shortly after delivery, however, bacteria enter the infant’s digestive tract, chiefly populating the colon and rectum—an essential event for immune maturation (12, 13). Thus, while sterile before birth, the intestine becomes colonized soon after delivery. This suggests that the intestinal villi and glands develop in a relatively sterile environment during fetal intestinal recanalization, and a locally dysregulated immune response may represent an important contributor to inflammation within the septal tissue. However, the potential molecular mechanisms involved in regulating this biological process remain unclear.

Currently, some studies have reported that the nuclear factor kappa B (NF-*κ*B) signaling pathway is a major regulator of inflammatory responses (14–16). It has also been involved in immunomodulatory responses during embryonic development and has been implicated in the development and progression of pediatric congenital anomalies, including biliary atresia (17–21), anorectal malformations (22), Hirschsprung’s disease (23), congenital necrotizing enterocolitis (24), and congenital diaphragmatic hernia (25). However, human septal specimens are very rare and difficult to obtain, and results from animal IA models or *in vitro* studies cannot accurately represent humans. The expression of NF-*κ*B signaling pathway components in the septal tissue of IA has not been reported.

In the present study, we determined the expression of NF-*κ*B signaling components in human septal tissue samples arising from type I IA by immunohistochemistry and explored the possible role of NF-*κ*B signaling in the formation and development of human septa.

## 2 Materials and methods

### 2.1 Materials and reagents

Phosphate-buffered saline (PBS) solution, 4% paraformaldehyde (PFA), ethanol, and sodium chloride were purchased from Sigma-Aldrich Ltd. (St. Louis, MO, USA). A light microscope and a digital camera (Olympus SZX7, Nikon, Eclipse E600, Japan) were used for tissue morphology. All slides were scanned using a high-quality digital scanner (Pannoramic Diagnostic Scanner, 3D HISTECH, Hungary) for analysis.

### 2.2 Patient and septum specimen collection

The intestinal segment with the septum was collected at the Department of Surgery of Beijing Children’s Hospital Heilongjiang Hospital, Capital Medical University, between September 2016 and December 2021. The information about the patients was the same as in our previous report (26). The tissue harvest method was the same as in our previous study (8, 26). In brief, the intestinal segment with the septum was surgically removed, followed by an end-to-end anastomosis, and adjacent proximal normal intestinal wall tissues near the incision were harvested and used as controls. The tissues were excised using surgical scissors rather than electrocautery to prevent thermal damage. Patients with IA did not undergo any other treatment prior to surgery. The use of human intestinal tissues for the study was approved by the Medical Ethics Committee of Beijing Children’s Hospital Heilongjiang Hospital, Capital Medical University (Ethics approval number: 2020-IEC-06), and the Medical Ethics Committee of Capital Institute of Pediatrics (Ethics approval number: SHERLL2022047). Written informed consent was obtained from the patients’ parents.

### 2.3 Immunohistochemistry

The method used for immunohistochemistry was the same as described previously (27–29). Briefly, sections of 5 μm thickness were deparaffinized and rehydrated, and endogenous peroxidases were quenched by incubation in 3% hydrogen peroxide in methanol for 5-10 min. Following antigen retrieval, the sections were incubated with a blocking solution containing 5% normal goat serum (Dako Bioresearch, USA) for 1 h at room temperature. The sections were then incubated with primary antibodies (Table 1) overnight at 4°C in the dark, washed in phosphate-buffered saline (PBS), and incubated with an HRP-conjugated secondary antibody (Santa Cruz Biotechnology, CA, USA) for 2 h. The sections were subsequently incubated with DAB (3,3ʹ-diaminobenzidine tetrahydrochloride) to visualize antibody binding and counterstained with hematoxylin.

**Table 1:** Primary monoclonal/polyclonal antibodies used in this study.

Antigen	Abbreviation	Dilution	Species	Manufacturer	Catalog number
Transforming growth factor-β-activated kinase 1	TAK1	1:200	Rabbit	Abcam	4564
I Kappa B Kinase-alpha/beta	IKK-α/β	1:300	Rabbit	Abcam	2697
Nuclear factor kB	P65 NF-*κ*B	1:300	Rabbit	Abcam	8242
Interleukin-6	IL-6	1:200	Rabbit	Proteintech	21865-1-AP
Tumor necrosis factor-alpha	TNF-α	1:400	Mouse	Proteintech	60291-1-Ig

### 2.4 Semiquantitative analysis of immunohistochemical expression

Immunohistochemistry staining was quantified using a double-blind assessment procedure. Four fields were randomly selected from each section at  ×200 magnification under a conventional light microscope. Brown-yellow granular staining in mucosal or submucosal glandular epithelial cells was considered positive immunoreactivity. Integrated optical density (OD) values and stained areas were measured using Image-Pro Plus 6.0, and mean optical density was calculated as integrated optical density divided by area.

### 2.5 Statistical analysis

Statistical analyses were performed using SPSS version 18.0. Continuous variables are expressed as mean ± standard deviation (SD). Differences between two groups were evaluated using an independent-samples *t*-test. A two-sided *p*-value <0.05 was considered statistically significant.

## 3 Results

### 3.1 Increased expression of TAK1, IKK-α/β, and P65 NF-*κ*B in the septum

In this study, a total of eight patients had prenatal MRI findings of small bowel dilatation, and after birth, they experienced postprandial abdominal distension and bilious vomiting. An ultrasound examination indicated IA. All patients were diagnosed with type I IA based on both observation during surgery and postoperative pathological diagnosis.

Immunohistochemistry showed weak transforming growth factor-β-activated kinase 1 (TAK1) expression in the cytoplasm of mucosal glandular epithelial cells of the normal intestinal wall, whereas its expression was markedly increased in the corresponding cells of septal tissue (Figure 1A). We next examined I kappa B kinase-alpha/beta (IKK-α/β) expression in the mucosal layers of the two groups and found that, compared with normal mucosa, septal mucosa exhibited a significantly increased level of cytoplasmic IKK-α/β (Figure 1B). Given that P65 NF-*κ*B is a downstream effector of the NF-*κ*B pathway, we further assessed its expression in the mucosal layers of both groups, and the results showed that compared with the normal epithelium, a markedly increased expression of P65 NF-*κ*B was noted in the septal epithelium (Figure 1C). A semiquantitative analysis indicated that the OD value of TAK1 was 0.040 ± 0.005 in the normal intestinal wall and 0.265 ± 0.030 in the septum (*p* = 0.0008), the OD value of IKK-α/β was 0.075 ± 0.010 in the normal intestinal wall and 0.340 ± 0.040 in the septum (*p* = 0.0006), and the OD value of P65 NF-*κ*B was 0.035 ± 0.005 in the normal intestinal wall and 0.270 ± 0.030 in the septum (*p* = 0.0008), further confirming that TAK1, IKK-α/β, and P65 NF-*κ*B were significantly increased in the mucosal glandular epithelium of septal tissue compared with the normal intestinal wall (Figure 2).

**Figure 1:**
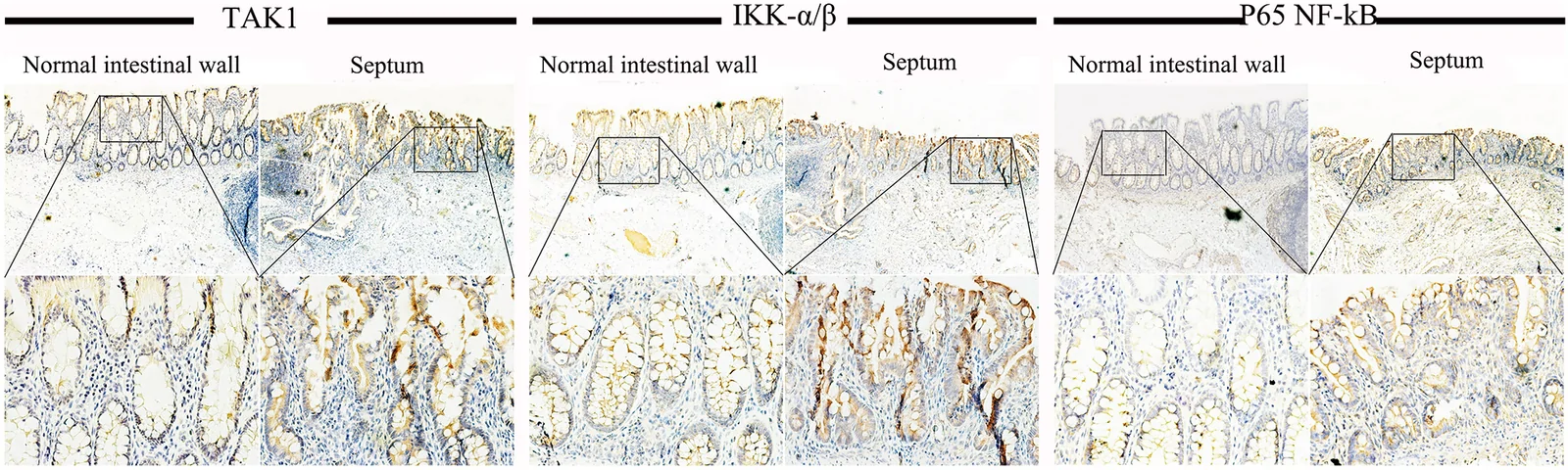
TAK1, IKK-α/β, and P65 NF-*κ*B expression in the mucosa of septa from type I IA and normal control under immunohistochemistry (upper panel, 100X; lower panel, 200X).

**Figure 2:**
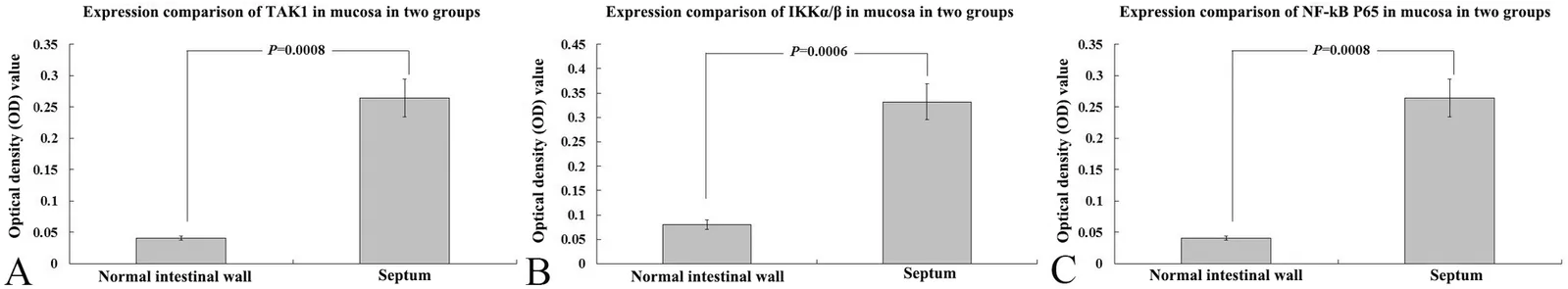
A semiquantitative comparison of TAK1, IKK-α/β, and p65 NF-κB expression in the mucosa of septa from patients with type I intestinal atresia (IA) and adjacent normal intestinal walls. **(A)** TAK1 expression; **(B)** IKK-α/β expression; **(C)** p65 NF-κB expression.

### 3.2 Increased expression of proinflammatory cytokines in septa

We further proceeded to assess the expression levels of the downstream proinflammatory cytokines of the NF-*κ*B signaling pathway in the two groups to confirm the activation of this pathway. Immunohistochemistry staining showed that the expression of tumor necrosis factor-alpha (TNF-α) and interleukin-6 (IL-6) in both the mucosal and the submucosal/muscular layers was significantly increased in septa compared with normal intestinal walls (Figure 3). The semiquantitative analysis indicated that in the mucosa, the OD value of TNF-α was 0.025 ± 0.005 in the normal intestinal wall and 0.072 ± 0.004 in the septum (*p* = 0.0003), while the OD value of IL-6 was 0.042 ± 0.005 in the normal intestinal wall and 0.055 ± 0.004 in the septum (*p* = 0.0165). In the muscular layer, the OD value of TNF-α was 0.013 ± 0.004 in the normal intestinal wall and 0.034 ± 0.007 in the septum (*p* = 0.0019), the OD value of IL-6 was 0.004 ± 0.001 in the normal intestinal wall and 0.046 ± 0.007 in the septum (*p* = 0.0009). The semiquantitative data further confirmed that TNF-α and IL-6 were significantly increased in the mucosal glandular epithelium of septal tissue compared with the normal intestinal wall (Figure 4).

**Figure 3:**
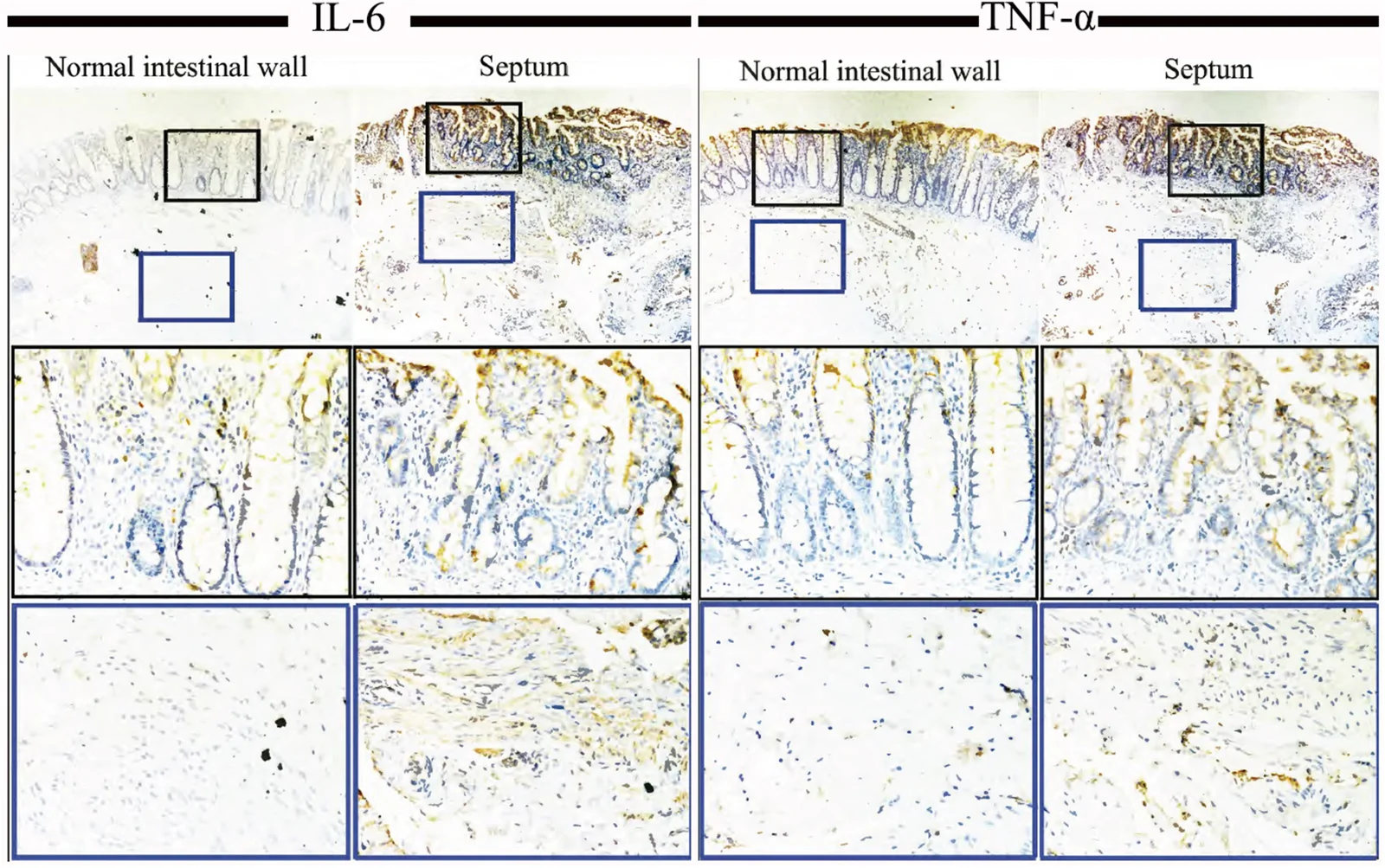
TNF-α and IL-6 expression in the mucosal and submucosal/muscular layers of the septum from type I IA and normal control under immunohistochemistry (upper panel, 40X; lower panel, 200X.).

**Figure 4:**
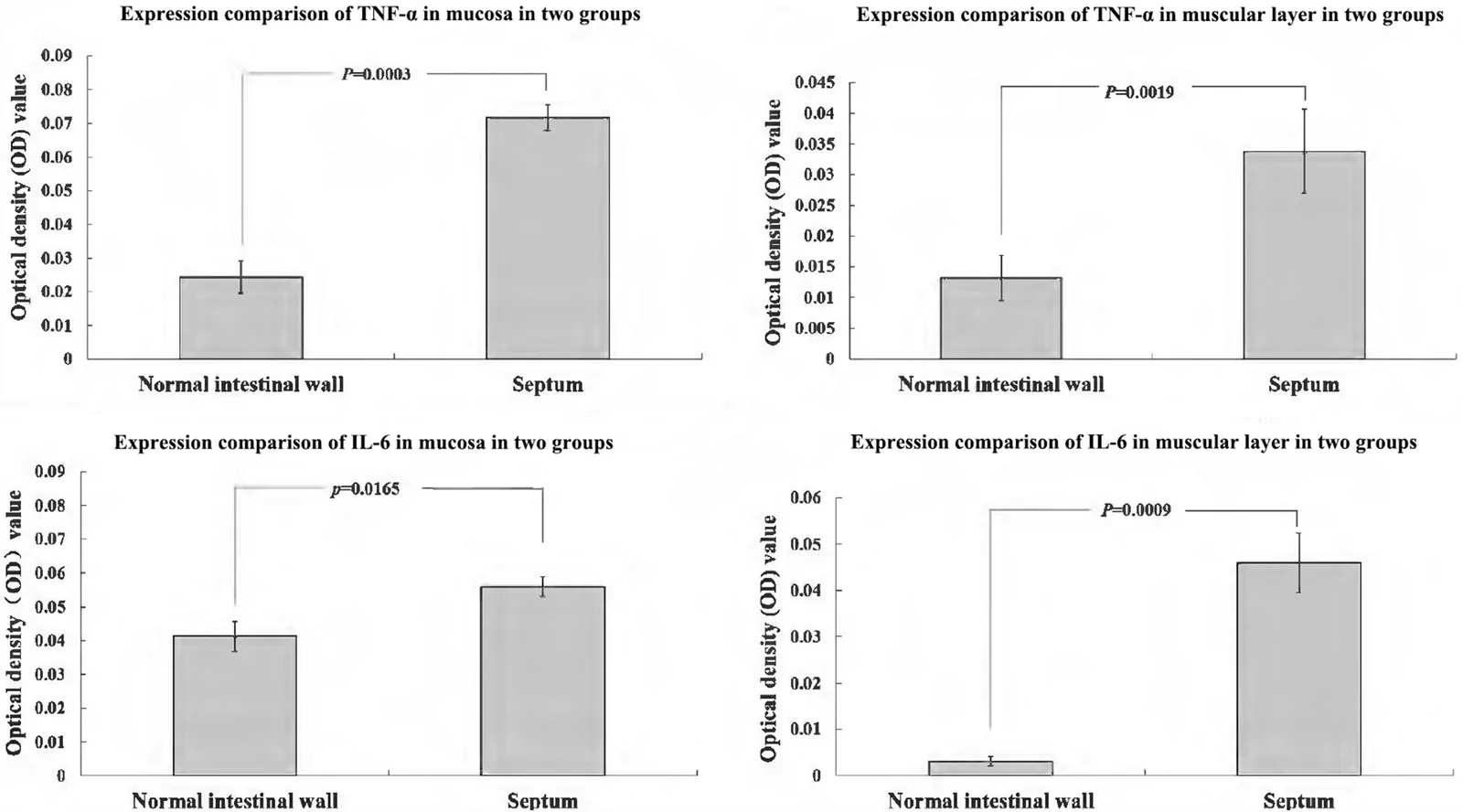
A semiquantitative comparison of TNF-α and IL-6 expression in septa and normal intestinal walls.

## 4 Discussion

The etiology of congenital small intestinal atresia remains unclear. In a previous study, we focused on the developing midgut during early human embryogenesis and characterized the morphology and differentiation of embryonic intestinal vacuoles (8). By comparing the extent of intestinal recanalization at different anatomical sites, we provided a preliminary developmental and morphological explanation for the progressively decreasing incidence of IA from the duodenum to the ileum (9). Based on these observations, we subsequently investigated the septa of IA and found a reduction in immune barrier-associated cells within the mucosal layer in septa compared with the normal intestinal wall (30, 31). These findings prompted us to consider the following possibility: During embryonic development, intestinal villi and glands are immersed in relatively sterile amniotic fluid and therefore remain in a largely aseptic environment. In the absence of an abnormal external immune response mediated by amniotic fluid-associated factors, intrinsic local immune dysregulation within the intestinal tissue may represent an important mechanism underlying the inflammatory response observed in septa. Regardless of which immune cell populations participate in this aberrant intrinsic immune response, molecular signaling pathways and their associated molecules within the mucosal layer of septa may play critical roles in mediating and regulating this biological process.

The NF-*κ*B signaling pathway is a major molecular pathway involved in inflammatory responses (32, 33). It is also an important intracellular transcriptional regulator implicated in the development and progression of congenital abnormalities in children (34, 35) and can also mediate abnormal tissue and cellular apoptosis during embryogenesis (36, 37). The NF-*κ*B family consists primarily of five members: p65(RelA), RelB, p50/p105, p52/p100, and c-Rel. Under resting conditions, intracellular p65 associates with p50 to form a heterodimer that binds to the inhibitor I*κ*B-α, remaining in an inactive complex. In response to inflammatory stimuli, I*κ*B kinase β (IKK-β) becomes phosphorylated and subsequently induces the phosphorylation of I*κ*B-α, resulting in the release of the p65/p50 heterodimer. The p65 then translocates to the nucleus, where it initiates the transcription and expression of target genes. In the present study, histological approaches were used to examine the expression of key molecules in this signaling pathway within the mucosal layers of normal intestinal wall tissue and septa, aiming to determine, at the histological level, whether abnormal expression of NF-*κ*B pathway–associated proteins occurs during the formation and progression of septa. Because the NF-*κ*B signaling pathway is a proinflammatory cascade whose activation leads to the enhanced expression of downstream inflammatory mediators, including TNF-*α* and IL-6 (38–40), we further investigated the expression of TNF-*α* and IL-6. The results demonstrated their increased expression and confirmed the activation of the NF-*κ*B signaling pathway.

To minimize experimental variability attributable to interindividual and potential interspecimen differences, adjacent proximal normal intestinal wall tissues from the septum were obtained from the same patient. The results showed that, in normal intestinal mucosa, key molecules of the NF-*κ*B signaling pathway, including the upstream molecule TAK1, the intermediate molecules IKK-α/β, and the downstream molecule p65 NF-*κ*B, were weakly expressed in the cytoplasm of mucosal glandular epithelial cells. In contrast, their expression was significantly increased in the cytoplasm of mucosal glandular epithelial cells within the septum. This suggests that abnormal expression of proteins within the NF-*κ*B signaling pathway may be involved in the pathogenesis of IA. Taken together with our previous observations of a reduction in immune barrier-associated cells within the mucosal layer in septa compared with the normal intestinal wall (30, 31) and the fact that these immune barrier-associated cells are developmentally derived from intestinal stem cells, we further propose that abnormalities in intestinal stem cell differentiation and their integration into the intestinal mucosa during embryonic development may be associated with aberrant signaling pathway activation. Therefore, the formation and progression of the septum may involve abnormalities not only in developmental signaling pathways but also in inflammatory signaling pathways. Because all specimens were obtained during postnatal surgery, the detected signals may not exclusively reflect embryonic alterations. To reduce mucosal inflammation attributable to postnatal intestinal and surgical factors, adjacent normal intestinal wall tissue from the same patient was used as a control alongside septal specimens to best capture the differences between septal and normal intestinal wall tissue within the same IA patient. Furthermore, due to the scarcity of IA specimens and challenges in their collection, this study had a limited sample size (*n* = 8), thereby constraining statistical power.

Nevertheless, the mechanism by which inflammatory responses contribute to the formation of the septum is unlikely to be mediated by a single signaling pathway. The NF-*κ*B signaling pathway represents only one downstream component of a complex inflammatory signaling cascade. Because IA is a relatively uncommon congenital gastrointestinal malformation causing neonatal intestinal obstruction, the collection of septal tissue specimens is inherently difficult, and accumulating sufficient samples remains a challenge. Consequently, the present study provides only preliminary histological evidence of NF-*κ*B pathway activation and abnormal protein expression in the septum. The expression patterns and roles of signaling pathways upstream of NF-*κ*B remain unclear. Furthermore, it remains to be determined whether activation of the downstream NF-*κ*B pathway is driven by a single upstream signaling pathway or results from the coordinated activation of multiple upstream pathways. In addition to NF-*κ*B signaling, whether other inflammatory signaling pathways participate in the formation and progression of the septum also requires further investigation. It is also unclear whether NF-*κ*B and other inflammatory signaling pathways are regulated by common upstream signals or by distinct regulatory mechanisms. Moreover, the relationships between developmental signaling pathways and NF-*κ*B signaling, as well as between developmental pathways and the upstream regulatory pathways controlling NF-*κ*B activation, remain to be elucidated. These questions constitute important targets for our future investigations.

In conclusion, we observed an increased expression of NF-*κ*B signaling pathway molecules within the septal mucosal layer of type I IA. These findings provide molecular evidence supporting the involvement of inflammatory signaling during impaired embryonic intestinal recanalization and identify potential targets for subsequent screening of additional signaling pathways and for investigating the regulatory interactions among these pathways.

## Data Availability

The raw data supporting the conclusions of this article will be made available by the authors, without undue reservation.
